# Ebola Hemorrhagic Fever Transmission and Risk Factors of Contacts, Uganda^1^

**DOI:** 10.3201/eid0911.030339

**Published:** 2003-11

**Authors:** Paolo Francesconi, Zabulon Yoti, Silvia Declich, Paul Awil Onek, Massimo Fabiani, Joseph Olango, Roberta Andraghetti, Pierre E. Rollin, Cyprian Opira, Donato Greco, Stefania Salmaso

**Affiliations:** * Istituto Superiore di Sanità, Rome, Italy; † St. Mary’s Hospital Lacor, Gulu, Uganda; ‡ Ministry of Health, Kampala, Uganda; § Centers for Diseases Control and Prevention, Atlanta, Georgia, USA

## Abstract

From August 2000 through January 2001, a large epidemic of Ebola hemorrhagic fever occurred in Uganda, with 425 cases and 224 deaths. Starting from three laboratory-confirmed cases, we traced the chains of transmission for three generations, until we reached the primary case-patients (i.e., persons with an unidentified source of infection). We then prospectively identified the other contacts in whom the disease had developed. To identify the risk factors associated with transmission, we interviewed both healthy and ill contacts (or their proxies) who had been reported by the case-patients (or their proxies) and who met the criteria set for contact tracing during surveillance. The patterns of exposure of 24 case-patients and 65 healthy contacts were defined, and crude and adjusted prevalence proportion ratios (PPR) were estimated for different types of exposure. Contact with the patient’s body fluids (PPR = 4.61%, 95% confidence interval 1.73 to 12.29) was the strongest risk factor, although transmission through fomites also seems possible.

Ebola hemorrhagic fever (EHF) is a severe viral disease caused by three of the four species of “Ebola-like viruses” (*1*), which are probably maintained in an as-yet-undefined natural reservoir in the rain forests of Africa (*2*). Epidemics occur when an infectious case-patient is introduced into a susceptible population. The first recognized epidemics occurred almost simultaneously in 1976 in southern Sudan (284 cases and 117 deaths) (*3*) and in a nearby region of the Democratic Republic of Congo (318 cases and 280 deaths) (*4*). A major mode of transmission was within hospitals, especially in the early stages of the outbreaks. Person-to-person transmission also occurred outside the hospital setting, with numerous community-acquired cases (*3*,*4*).

In 1995, another large epidemic occurred in Kikwit, in the Democratic Republic of Congo, with 315 cases and 244 deaths (*5*). The primary mode of transmission was person-to-person transmission to household members who had had direct contact with sick persons or their body fluids, especially during the late stage of the disease (*6*). However, the source of infection remained unknown for 12 case-patients, which led to the suspicion that the virus was transmitted by airborne particles or fomites (*7*).

The largest epidemic (425 presumptive cases and 224 deaths) occurred from August 30, 2000 (i.e., the earliest presumptive case), to January 9, 2001 (i.e., onset of the last case), in the Republic of Uganda, which borders both the Democratic Republic of Congo and Sudan (*8*–*11*). Since then, epidemics have been occurring with increasing frequency. Specifically, between December 2001 and March 2002, outbreaks occurred in the Republic of Gabon (65 cases and 53 deaths) (*12*,*13*) and in the neighboring Republic of Congo (57 cases and 43 deaths) (*12*). In February 2003, cases again began to be reported in the Republic of Congo, where 13 laboratory-confirmed case-patients and 127 epidemiologically linked case-patients, including 123 deaths, have been reported to date (*14*).

During the epidemic in Uganda, a national task force, in collaboration with an international team of health professionals, conducted activities for controlling the epidemic and managing cases (*11*). The area in which the epidemic was mainly concentrated was the Gulu District, a savannah area located in the north and mainly inhabited by Nilotic tribes. Most of the district’s 400,000 inhabitants live in Gulu Town or in one of several camps, located in rural areas, for persons who have been internally displaced because of the insecurity caused by the activity of insurgents.

On October 8, an outbreak of EHF was suspected at St Mary’s Hospital Lacor (hereafter termed Lacor Hospital), a nonprofit facility located several kilometers from Gulu Town. Two days later, isolation wards were set up in the district’s major hospitals, i.e., Lacor Hospital and the Gulu Government Hospital. In Lacor Hospital, only the hospital staff provided patient care in the isolation ward, whereas in the Gulu Government Hospital, relatives were allowed to contribute, which is the usual practice in Ugandan hospitals. The staff of both hospitals adopted strict barrier nursing precautions (e.g., gloves, masks, gowns, aprons, rubber boots); in the Gulu Government Hospital, these precautions were partially extended to patients’ relatives. On October 15, the outbreak was confirmed, and a system of daily case reporting, including a computerized database, was established. A case-patient was defined as a person who experienced at least one of the following events (*9*,*10*): 1) unexplained bleeding; 2) abrupt onset of fever and three or more of the following symptoms or signs: headache, vomiting, anorexia, diarrhea, weakness, or severe fatigue, abdominal pain, body aches or joint pain, difficulty in swallowing, difficulty in breathing, and hiccups; and 3) death from unexplained causes.

On October 21, 2000, the Centers for Disease Control and Prevention (CDC) set up a laboratory for performing enzyme-linked immunosorbent assays (ELISAs) for Ebola antigens and antibodies and reverse transcriptase-polymerase chain reaction (RT-PCR) at Lacor Hospital. Laboratory confirmation (positive result for Ebola virus antigen or Ebola immunoglobulin [Ig] G antibody) was obtained for 218 (51.3%) of the total 425 presumptive cases involved in the epidemic (*9*,*10*).

At approximately the same time, a surveillance system for contact tracing and case finding was established. A contact was defined as a person who had at least one of the following exposures: 1) physical contact with a case-patient, alive or dead; 2) slept in the same hut or house with a case-patient during the disease period; 3) contact with a case-patient’s body fluids during the disease period; and 4) contact with a case-patient’s linens or other possible fomites during the disease period and just after death. Members of the surveillance teams and the hospital staff were not considered contacts, even if they were exposed to a case-patient, because they had been taught how to protect themselves. For each case-patient, a list of contacts was created; all contacts were followed by daily home visits for 21 days (maximum incubation period) from the last contact with the case-patient (*11*).

In November and December 2000, we collected additional data from a group of contacts (or their proxies) concerning the nature and timing of their exposure to case-patients. Our objective was to trace chains of transmission and identify risk factors for transmission among a group of exposed persons in the community. This study, the results of which are reported here, was fully integrated into the surveillance activities described above and was authorized by the director of the Gulu District Health Services and the Ugandan Ministry of Health.

## Methods

### Study Design and Population

To retrospectively trace the chain of transmission, we interviewed three laboratory-confirmed case-patients in the Lacor Hospital who had onset of symptoms October 23–28 (referred to as “study case-patients”; see Table 1 for other definitions). We asked them to identify the persons from whom they had probably acquired the disease (referred to as index patients). In turn, the index patients (or their next of kin living in the same village if they had died, as was usually the case) were then asked to identify the persons from whom they had probably acquired the disease (also referred to as index patients). This process was repeated until we reached the patients whose source of infection could no longer be identified (referred to as primary case-patients). For each of the index patients and primary case-patients, we then reviewed the list of persons with whom they had been in contact since the onset of their symptoms; such information had been routinely collected as part of surveillance. Then, as 21 days had passed since the last exposure, each name on the list was matched with a name on the list of reported case-patients in order to identify the contacts in whom the disease had developed (collateral case-patients). The process was then repeated prospectively with the collateral case-patients for as many generations as possible.

**Table 1:** Definitions used in the chain of transmission of Ebola hemorrhagic fever

Classification	Definition Crude RR(95%CI) P-value
Study patients	The three laboratory-confirmed case-patients from whom we retrospectively identified the other case-patients and their contacts
Index patients	The nine case-patients retrospectively identified from the study patients as the source of infection (including primary case-patients)
Primary case-patients	The three earliest patients for whom we were not able to identify the source of infection
Collateral case-patients	Cases generated by the index patients, or by other collateral case-patients, and identified by matching the list of contacts of these persons with the list of reported cases
Postprimary case-patients	Case-patients for whom we were able to identify the source of infection
Contacts	Persons exposed to a case-patient, listed by the surveillance teams using the definition reported in the background section.
Healthy contacts	Contacts in whom the disease did not develop within 21 days of the last exposure

To identify risk factors, we interviewed all of the identified contacts (or their proxies) of the primary, index, and collateral case-patients, irrespective of their status (patient or healthy contact). To this end, we developed a questionnaire that focused on the exact type and timing of exposure to index patients.

### Data Analysis and Statistical Methods

We performed univariate analyses to evaluate the strength of associations between the different types of exposure and disease, by comparing disease prevalence among persons with a given exposure to that among persons without that exposure and by testing the resulting differences with the chi-square test or, when appropriate, the Fisher exact test. Those risk factors independently associated with the disease were evaluated in multivariate analyses by using log-binomial regression models after we ascertained the absence of a significant multiple colinearity among the variables. The crude and adjusted prevalence proportion ratios (PPR) and their 95% confidence intervals (CI) were used to describe the strength of the associations (*15*).

## Results

### Chains of Transmission

The Figure illustrates the three reconstructed chains of transmission; each consisted of three identified generations of cases (excluding the study case-patients). The 27 identified case-patients consisted of, in addition to the 3 laboratory-confirmed patients with whom we began the study, 9 index case-patients (including 3 primary case-patients, all young women whose source of infection was unknown), and 15 collateral case-patients. Of the 24 postprimary patients, 14 (58.3%) lived in the Gulu Town or Municipality, and 10 (41.7%) lived in rural areas of the Gulu District. One patient was a newborn, and three were infants. The remaining 20 patients (83.3%) ranged in age from 14 to 70 years; 14 (70.0%) of these 20 patients were female, and most were housewives or subsistence farmers (70.0%).

**Figure:**
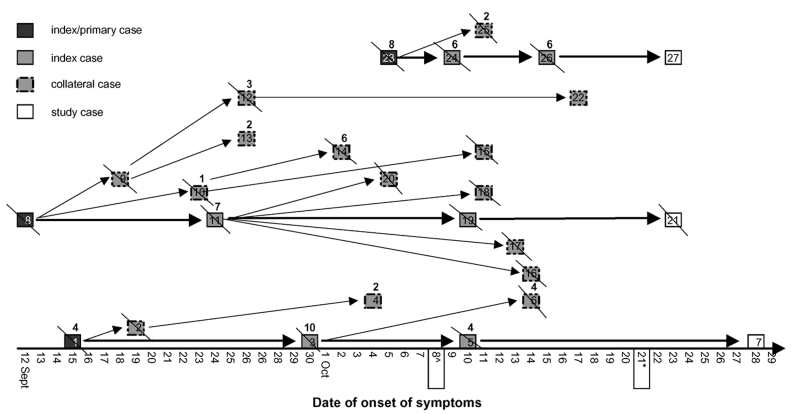
Chains of transmission relative to 27 Ebola cases, Gulu District, Uganda (September–October 2000). The numbers above the blocks indicate the total number of healthy contacts identified for that patient. The slashes indicate patients who died. The isolation ward was opened on October 8. *A laboratory facility for serologic diagnosis of Ebola was set up at Lacor Hospital by the Centers for Disease Control and Prevention on October 21. Description of individual cases follows: 1. AF, young woman, admitted at Gulu Hospital Sept. 19, died the same day. She was buried the following day, next to her parents’ house, without known identified case-patients among those who attended the burial ceremony; 2. OS, son of AF (4 months old); breastfed even during the last days of his mother’s life; admitted to Lacor Hospital; died Sept. 30. 3. OA, father-in-law of AF; nursed his daughter-in-law during the last days of the disease, both at home and in the hospital; reported contact with blood and vomit; died Oct. 7. 4. AR, grandmother of OS; nursed the child after the mother’s death; reported contact with feces and urine; survived. 5. ON, cousin of OA, whom ON touched before and after OA’s death; no reported contacts with body fluid; died Oct. 16. 6. OR, brother of OA; nursed him during last days; reported contacts with feces; died Oct. 17. 7. OJ, brother of ON; used the blanket left by his brother; survived. 8. AE, young woman who sold beer to soldiers; died Sept. 17. 9. OS, son of AE; breastfed; died Sept. 21. 10. AD, mother of AE; nursed her; reported contact with vomit and feces; prepared the dead body; died Oct. 1. 11. AJ, sister of AE; nursed her; reported contact with feces and vomit; prepared the dead body; died Oct. 4. 12. AV, aunt of OS; nursed the child after the mother’s death; reported contact with vomit and feces; died Oct. 11. 13. AN, cousin of OS; they slept together; reported contact with vomit and feces; survived. 14. AV, daughter of AD; nursed her; reported contact with vomit and feces; died Oct. 7. 15. AV, niece of AD; reported direct contact with her during illness; died Oct. 23. 16. AS, daughter of AJ; nursed her, both at home and in the hospital; no reported contact with body fluids; died Oct. 24. 17. AS, co-wife of A J; nursed her; reported contact with blood; died Oct. 24. 18. AE, sister-in-law of AJ; assisted her during delivery on Sept. 28; died Oct. 17. 19. LV, aunt of AJ; assisted her during delivery on Sept. 28; died Oct. 22. 20. OW, son of AJ; born on Sept. 28; died Oct. 9. 21. OJ, husband of LV; nursed her; died Nov. 1. 22. AC, cousin of AV; nursed her; reported contact with feces; survived. 23. AG, young woman; lived next to the barracks; died Oct. 8. 24. AL, sister of AG; nursed her; reported contact with vomit; died Oct. 18. 25. JB, sister of AG; nursed her; reported contact with vomit; died Oct. 26. OR, son of AL; breastfed; died Oct. 21. 27. AF, grandmother of OR; nursed the child after the mother’s death; reported contact with feces and vomit; survived.

The 24 postprimary patients had onset of symptoms from September 18 to October 28, 2000. The incubation period (i.e., time elapsed between either the last or the first contact with the index patient and the onset of symptoms) was 1–16 days (median 6 days), when the last contact was considered, and 1–12 days (median 12 days), when the first contact was considered. All three infants had an incubation period of <7 days.

Twenty (83.3%) of the 24 postprimary case-patients were admitted to the hospital;13 (65.0%) were admitted after the isolation ward had been created. The four patients not admitted to the hospital (a newborn, two infants, and an elderly woman) died within 3 to 11 days of disease onset. Of the 20 hospital patients, 7 were still in the hospital when the laboratory was set up, and 3 were admitted afterwards; all 10 of these patients tested positive for Ebola antigens, IgG, or both.

Of the 20 hospitalized patients, 15 died. Among these 15 patients, the duration of illness (from onset of symptoms to death) was 3–15 days (median 10 days); the duration of hospitalization (from admission to death) was 2–11 days (median 5 days). Among the five surviving patients, the duration of illness (from onset of symptoms to discharge upon clinical recovery) was 10–25 days (median 15 days); the duration of hospitalization was 8–22 days (median 13 days).

Of the 27 patients, all of the primary and secondary case-patients died. Of the remaining 17 patients, 12 (70.6%) died. Of the four persons who died without being admitted to the hospital, two had secondary cases and two had tertiary cases.

In the legend to the Figure, the 27 cases are briefly described and the mode of transmission is summarized for the 24 postprimary cases. The newborn (case-patient 20) was delivered by a sick woman 4 days after the onset of symptoms, and the other three infants (case-patients 2, 9, and 26) had been breastfed by sick mothers. The other 20 postprimary cases were all members of the extended family (household contacts) of the case-patients to whom they had been exposed. All but one (95%) had had direct physical contact with the patient who was the likely source of their disease; the remaining person (case-patient 7) had slept wrapped up in a blanket left by his brother, who had just died of EHF.

Among the 20 postprimary case-patients who were >14 years of age, 15 (75.0%) reported that they had been exposed to the body fluids of their index patient; 11 (55.0%) had washed the index patient’s clothes; and 18 (90.0%) had taken care of the index patient at some point during his or her illness. Twelve of these 18 persons had taken care of the index patient until death, either in the hospital (n = 6) or at home (n = 6). Eleven (55.0%) of these 20 postprimary patients had slept in the same hut or house as the index patient; of these, 5 had slept with the index patient on the same mat or mattress. Six (30.0%) of these 20 postprimary patients had shared meals with index patients (picking up food with their fingers from the same plate). Sixteen (80.0%) of the 20 adult postprimary patients had attended the funeral of their index patient; 11 had also prepared the body for the ceremony or simply touched the dead body; 11 had participated in the communal meal during the ceremony; and 7 had participated in the ritual handwashing during the ceremony.

### Healthy Contacts

We also interviewed the 65 apparently healthy contacts of the 9 index patients and 15 collateral case-patients. Notably, not all patients generated contacts, and the six who did not were all third- (n = 5) or fourth-generation case-patients. Five had had onset of symptoms after the isolation ward was created.

Of the 65 healthy contacts, 39 (60.0%) lived in the Gulu Municipality and 23 (35.4%) in rural areas of the Gulu District; information on residence was not available for the remaining three. Two of the healthy contacts (3.1%) were infants, and four (6.2%) were 3–8 years of age The remaining 59 (90.8%) ranged in age from 10 to 70 years; 33 (55.9%) were female; most were housewives or subsistence farmers (60.0%).

One of the two infants had been separated from his sick mother early in the course of the mother’s illness; the other infant had been breastfed during his mother’s illness. All four of the children 3–8 years of age had slept in the same hut as their sick parent and had had direct physical contact with their sick parent or relative (none of them had taken care of the sick person). None of these four children was reported to have been in contact with the patient’s body fluids.

Of the 59 healthy contacts >10 years of age, 50 (84.7%) were extended family members of the patient (household contacts); 9 were neighbors of the patient. Forty-seven (79.7%) had had direct physical contact with the case-patient; 15 (25.4%) had been exposed to body fluids; 18 (30.5%) had washed the patient’s clothes; and 25 (42.4%) had taken care of the sick person. Of these 25 persons, 11 had taken care of their relative up to the last days of life, either in the hospital (n = 8) or at home (n = 3). Moreover, 13 (22.0%) had slept in the same hut as the patient; 4 had shared the same mat; 7 (11.9%) had shared meals with the index patient (picking up food with their fingers from the same plate).

Thirty-seven (62.7%) of these 59 healthy contacts had attended the funeral of the patient; 14 of them had also touched the dead body. In addition, 14 healthy contacts had participated in the communal meal during the ceremony, and 9 had participated in the ritual handwashing.

### Risk Factors

Because of their particular exposures, infants <2 years were excluded from the analysis of risk factors. Among the 83 remaining contacts, disease developed in 20. Sixty-three contacts remained healthy. Among contacts, neither age (>30 years vs. ≤30 years: PPR = 1.38, 95% CI 0.64 to 2.97) nor sex (women vs. men: PPR = 1.54, 95% CI 0.66 to 3.60) was significantly associated with the disease (Table 2).

**Table 2:** Univariate analysis of risk factors for Ebola hemorrhagic fever among a population of 83 contacts, Gulu, Uganda, 2000

Risk factors	No. of cases (%)	Crude PPR (95% CI)^a^	p value	
**Demographic characteristics**				
Sex				
Male	6 (18.2)	1		
Female	14 (28.0)	1.54 (0.66 to 3.60)	0.446	
Age group (y)				
<30	9 (20.5)	1		
>30	11 (28.2)	1.38 (0.64 to 2.97)	0.571	
**Direct transmission**				
Touched sick person				
No	1 (7.7)	1		
Yes	19 (27.1)	3.53 (0.52 to 24.11)	0.173	
Touched body of deceased person				
No	9 (17.6)	1		
Yes	11 (34.4)	1.95 (0.91 to 4.17)	0.141	
Contact with body fluids				
No	5 (9.4)	1		
Yes	15 (50.0)	5.30 (2.14 to 13.14)	< 0.001	
**Indirect transmission**				
Shared meals				
No	14 (20.6)	1		
Yes	6 (40.0)	1.94 (0.89 to 4.22)	0.178	
Washed clothes				
No	9 (18.8)	1		
Yes	11 (31.4)	1.68 (0.78 to 3.60)	0.283	
Slept in the same hut/on the same mat				
No	9 (16.4)	1		
Shared only the hut	6 (35.3)	2.16 (0.90 to 5.19)		
Shared also the same mat	5 (45.5)	2.78 (1.15 to 6.70)	0.019 (for trend)	
Ritual handwashing during funeral				
No	13 (19.4)	1		
Yes	7 (43.7)	2.25 (1.08 to 4.72)	0.054	
Communal meal during funeral				
No	9 (15.5)	1		
Yes	11 (44.0)	2.84 (1.35 to 5.98)	0.012	

^a^PPR, prevalence proportion ratios; CI, confidence interval.

Contact with body fluids showed a strong association (PPR = 5.30, 95% CI 2.14 to 13.14). Persons who had had direct physical contact with a sick person were more likely to have acquired the disease (PPR = 3.53, 95% CI 0.52 to 24.11), as were those who had touched the body of the deceased person (PPR = 1.95, 95% CI 0.91 to 4.17), although these associations were not statistically significant.

Regarding indirect transmission, sleeping on the same mat (PPR = 2.78, 95% CI 1.15 to 6.70), participating in the ritual handwashing during the funeral ceremony (PPR = 2.25, 95% CI 1.08 to 4.72), and sharing a communal meal during the funeral ceremony (PPR = 2.84, 95% CI 1.35 to 5.98) were significantly associated with disease. Although the differences were not statistically significant, sharing meals, washing clothes, and sleeping in the same hut were associated with a higher risk of acquiring the disease.

In general, having taken care of a sick person represented a strong risk factor, although the level of risk was lower for persons who had provided care only at the early stage of the disease (PPR = 6.00, 95% CI 1.33 to 27.10), followed by the risk for those who provided care until the index patient’s death, either at the hospital (PPR = 8.57, 95% CI 1.95 to 37.66) or at home (PPR = 13.33, 95% CI 3.20 to 55.59) (Table 3).

**Table 3:** Univariate analysis of risk factors for Ebola hemorrhagic fever related to patient care and the number of types of direct contact among 83 contacts, Gulu, Uganda, 2000

Risk factors	No. cases (%)	Crude PPR (95%CI)^a^	p value
**Cared for patient**			
No	2 (5.0)	1	
Cared only during patient’s early stage	6 (30.0)	6.00 (1.33 to 27.10)	
Cared until the patient’s death at hospital the hospital	6 (42.9)	8.57 (1.95 to 37.66)	
Cared until the patient’s death at home	6 (66.7)	13.33 (3.20 to 55.59)	<0.001 (for trend)
**Number of types of direct contact**			
No direct contact	1 (16.7)	1	
One type of direct contact	1 (2.9)	0.18 (0.01 to 2.45)	
Two types of direct contact	10 (32.3)	1.94 (0.30 to 12.44)	
Three types of direct contact	8 (66.7)	4.00 (0.64 to 25.02)	<0.001 (for trend)

^a^PPR, prevalence proportion ratios; CI, confidence interval.

The risk tended to increase with the increasing number of different types of direct contact (chi square for trend p<0.001); the risk was higher among persons who were exposed through two (PPR = 1.94, 95% CI 0.30 to 12.94) or three different types of direct contact (PPR = 4.00, 95% CI 0.64 to 25.02), compared with the risk for those who had no direct contact (Table 3).

Factors related to direct and indirect transmission were analyzed separately in multivariate analyses (Table 4). The first model (i.e., factors related to direct transmission) showed that having had contact only with body fluids (adjusted PPR = 4.61, 95% CI 1.73 to 12.29) was strongly associated with the disease, whereas having only touched the patient during illness was not (adjusted PPR = 1.56, 95% CI 0.19 to 13.04). (The weak association found in the univariate analysis was probably confounded by contact with the patient’s body fluids.) Having touched the body of the deceased person (adjusted PPR = 1.84, 95% CI 0.95 to 3.55) showed a borderline significant association.

**Table 4:** Multivariate analyses on risk factors for Ebola hemorrhagic fever related to direct and indirect transmission among 83 contacts, Gulu, Uganda, 2000

Risk factors	Adjusted PPR^a^	95% CI^b^	p value
**Model 1: Direct transmission**			
Touching patient during illness	1.56	0.19 to 13.04	0.679
Touching dead body	1.84	0.95 to 3.55	0.069
Contact with patient fluid	4.61	1.73 to 12.29	0.002
**Model 2: Indirect transmission** ^c^			
Sharing meals	1.69	1.00 to 2.85	0.050
Washing clothes	1.02	0.47 to 2.22	0.957
Sleeping in the same hut/on the same mat			
Sharing only the hut	2.34	1.13 to 4.84	0.022
Sharing also the mat	2.93	1.16 to 7.38	0.023
Ritual handwashing during funeral	1.16	0.54 to 2.49	0.706
Communal meal during funeral	1.50	0.98 to 2.28	0.060

^a^PPRs, prevalence proportion ratios adjusted for all the variables included in the model. ^b^CI, confidence intervals. ^c^Model 2 has been run controlling for the potential confounding effect due to the intensity of direct contacts with a case-patient (less than two types of direct contacts versus two or more types of direct contacts).

The second model (i.e., factors related to indirect transmission and controlled for the potential confounding effect attributed to the number of different types of direct contact) showed that sleeping in the same hut (adjusted PPR = 2.34, 95% CI 1.13 to 4.84) and sleeping on the same mat (adjusted PPR = 2.93, 95% CI 1.16 to 7.38) were independent risk factors. However, weak associations were found for sharing meals with a sick person and participating in the communal meal during the funeral, whereas the ritual handwashing during the funeral and washing the sick person’s clothes were not risk factors.

## Discussion

Although the number of EHF epidemics in sub-Saharan Africa has been increasing and EHF viruses have recently been classified as agents that could be used as possible biological weapons (*16*), epidemiologic data on the modalities of transmission are still limited (*6*) because of the sporadic and sudden nature of outbreaks. In the Ugandan outbreak, the hospital isolation wards have been important in managing cases. This fact was demonstrated by the finding that the patients with onset of symptoms after the institution of these wards on October 10 were the only ones who did not generate contacts, with the exception of an infant born on September 28, who had onset of symptoms on October 5 and died on October 9. Moreover, the higher death rate observed among primary and secondary case-patients (100%), in contrast with that among the most recent case-patients (70.6%), could be explained by the treatment provided in the hospital, though this treatment was mainly supportive.

The reconstruction of the chains of transmission was straightforward for three generations of case-patients, which suggests that person-to-person transmission occurred. Nevertheless, the source of infection of the primary patients remained unknown, although transmission was occurring in the community. As described in the Figure, most of the links in the chain of transmission were deceased; for this reason, most interviews were administered to proxies. Thus, the possibility that a nonhuman natural reservoir may have been involved could not be excluded.

Among the postprimary case-patients, the most important risk factor was direct repeated contact with a sick person’s body fluids, as occurs during the provision of care. As expected, the risk was higher when the exposure took place during the late stage of the disease at home. The risk was reduced when the patient stayed in a hospitals, probably because of the use of gloves, even before strict barrier nursing was implemented (*6*,*7*).

By contrast, simple physical contact with a sick person appears to be neither necessary nor sufficient for contracting EHF. In fact, one person in whom the disease developed was probably infected by contact with heavily contaminated fomites (patient 7), and many persons who had had a simple physical contact with a sick person did not become infected.

Transmission through contaminated fomites is apparently possible. In fact, the association found for having slept on the same mat or having shared meals with a sick person or with funeral participants remained after controlling for direct contact. However, having washed the clothes of a sick person and having participated in the ritual handwashing during the funeral ceremony were not significant risk factors.

Finally, although we cannot exclude the possibility of airborne transmission, this mode probably plays a minor role, if any. In fact, the association between having slept in the same hut and acquiring the disease was weak and could have been produced by some unidentified confounding variables. Furthermore, the reported Ebola virus aerosol transmission among nonhuman primates (*17*,*18*) has been demonstrated in laboratory experiments, which may be irrelevant in the natural context.

Studies conducted during outbreaks cannot be planned in advance, and the sample size is not predetermined, often resulting in a low statistical power for detecting significant associations between exposure and disease. In our study, the sample size (n = 83) reached a statistical power equal to 31% to detect as significant a PPR >2 (a total sample size of 275 would be needed to reach a statistical power equal to 80%).

Asking case-patients to identify the persons from whom they had probably acquired the disease could have introduced a bias because it implied a preconceived idea about how the infection was transmitted. However, most of the case-patients indicated that sick relatives whom they had cared for were their index patients; moreover, all of these index patients who were tested had positive results for Ebola antigens or antibodies. Both of these facts suggest that this bias did not significantly affect the results. Moreover, the retrospective design of the study, conducted in an emergency situation and in part based on a surveillance system that was created only in the third week of October, may have made it easier to identify sick contacts, as opposed to healthy contacts, because of the effect of recall bias. For this reason, we decided not to calculate the secondary attack rate or the reproductive rate. Furthermore, the group of uninfected contacts may have been inadvertently selected in a biased manner. Finally, the fact that the information was in most cases obtained from proxies may have also affected the results regarding risk factors. However, these results contribute to the knowledge on the patterns of transmission and risk factors for EHF, which is fundamental for better controlling new outbreaks.

The results of this study stress the importance of early detection and isolation of EHF patients in hospitals and the use of strict barrier nursing precautions in successfully controlling EHF outbreaks. Our findings on risk factors can also contribute to the efforts for educating communities and preventing the spread of the disease.
